# Low-dose total body irradiation facilitates antitumoral Th1 immune responses

**DOI:** 10.7150/thno.61459

**Published:** 2021-06-16

**Authors:** Dominik Sonanini, Christoph M. Griessinger, Barbara F. Schörg, Philipp Knopf, Klaus Dittmann, Martin Röcken, Bernd J. Pichler, Manfred Kneilling

**Affiliations:** 1 Werner Siemens Imaging Center, Department of Preclinical Imaging and Radiopharmacy, Eberhard Karls University Tuebingen, Germany.; 2 Department of Medical Oncology and Pneumology, Internal Medicine VIII, Eberhard Karls University Tuebingen, Germany.; 3 Division of Radiobiology and Molecular Environmental Research, Department of Radiation Oncology, Eberhard Karls University Tuebingen, Germany.; 4 Department of Dermatology, Eberhard Karls University Tuebingen, Germany.; 5 Cluster of Excellence iFIT (EXC 2180) "Image-Guided and Functionally Instructed Tumor Therapies", Eberhard Karls University Tuebingen, Germany.; 6 German Cancer Consortium (DKTK) and German Cancer Research Center (DKFZ), Heidelberg, Germany.

**Keywords:** Total body irradiation, cancer immunology, T helper cells, combined immunotherapy, RIP1-Tag2

## Abstract

CD4^+^ T helper cells are capable of mediating long-term antitumoral immune responses. We developed a combined immunotherapy (COMBO) using tumor antigen-specific T helper 1 cells (Tag-Th1), dual PD-L1/LAG-3 immune checkpoint blockade, and a low-dose total body irradiation (TBI) of 2 Gy, that was highly efficient in controlling the tumor burden of non-immunogenic RIP1-Tag2 mice with late-stage endogenous pancreatic islet carcinomas. In this study, we aimed to explore the impact of 2 Gy TBI on the treatment efficacy and the underlying mechanisms to boost CD4^+^ T cell-based immunotherapies.

**Methods:** Heavily progressed RIP1-Tag2 mice underwent COMBO treatment and their survival was compared to a cohort without 2 Gy TBI. Positron emission tomography/computed tomography (PET/CT) with radiolabeled anti-CD3 monoclonal antibodies and flow cytometry were applied to investigate 2 Gy TBI-induced alterations in the biodistribution of endogenous T cells of healthy C3H mice. Migration and homing properties of Cy5-labeled adoptive Tag-Th1 cells were monitored by optical imaging and flow cytometric analyses in C3H and tumor-bearing RIP1-Tag2 mice. Splenectomy or sham-surgery of late-stage RIP1-Tag2 mice was performed before onset of COMBO treatment to elucidate the impact of the spleen on the therapy response.

**Results:** First, we determined a significant longer survival of RIP1-Tag2 mice and an increased CD4^+^ T cell tumor infiltrate when 2 Gy TBI was applied in addition to Tag-Th1 cell PD-L1/LAG-3 treatment. In non-tumor-bearing C3H mice, TBI induced a moderate host lymphodepletion and a tumor antigen-independent accumulation of Tag-Th1 cells in lymphoid and non-lymphoid organs. In RIP1-Tag2, we found increased numbers of effector memory-like Tag-Th1 and endogenous CD4^+^ T cells in the pancreatic tumor tissue after TBI, accompanied by a tumor-specific Th1-driven immune response. Furthermore, the spleen negatively regulated T cell effector function by upregulation PD-1/LAG-3/TIM-3 immune checkpoints, providing a further rationale for this combined treatment approach.

**Conclusion:** Low-dose TBI represents a powerful tool to foster CD4^+^ T cell-based cancer immunotherapies by favoring Th1-driven antitumoral immunity. As TBI is a clinically approved and well-established technique it might be an ideal addition for adoptive cell therapy with CD4^+^ T cells in the clinical setting.

## Introduction

CD4^+^ T helper cells play a pivotal role in antitumoral immunity. Depending of their polarization, CD4^+^ T cells are capable of either suppressing antitumoral immune responses or inducing tumor regression 1. Unlike the direct cytotoxic ability of CD8^+^ T lymphocytes (CTLs) that have long been considered the primary population to mediate tumor rejection in Immune Checkpoint blockade (ICB) and adoptive cell therapy (ACT), the antitumoral potency of CD4^+^ T cells is mainly mediated by initiating macrophage polarization, natural killer (NK) cell recruitment, and cross priming to CTLs 2-4. Thus, employing CD4^+^ T cells and modifying their immunomodulatory properties have gained awareness, aiming to achieve higher immunotherapy response rates and long-term survival of cancer patients 5.

Previously, we developed a highly efficient ACT with IFN-γ producing CD4^+^ Tag2-specific T cells (Tag-Th1) in combination with a low-dose TBI of 2 Gy in an endogenous multistage insulin-producing pancreatic islet carcinoma RIP1-Tag2 mouse model 6. This combinatory immunotherapy doubled the life span of mice through the induction of a profound IFN-γ and TNF dependent tumor senescence 7. More recently, we demonstrated that addition of dual ICB with programmed death ligand 1 (PD-L1) and lymphocyte activation gene-3 (Lag-3) monoclonal antibodies (mAbs) to the Tag-Th1 cell and 2 Gy TBI treatment was applicable to control tumor burden even in late-stage RIP1-Tag2 mice with heavily progressed and metastasized carcimonas 8, 9; but the role of TBI in the low-dose of 2 Gy remained completely unclear.

TBI has been approved for clinical applications for many years; however, it is restricted almost exclusively to conditioning prior to autologous and allogenic stem cell transplantation 10-13. In contrast to standard conditioning regimens prior ACT, low-dose TBI is capable of modulating the immune system by provoking moderate lymphodepletion and inflammatory processes, both of which are generally attributed to low intensity irradiation therapy 14-20. Dudley *et al.* employed additive TBI to nonmyeloablative chemotherapy before adoptive transfer of *ex vivo* expanded tumor-infiltrating lymphocytes in patients with metastatic melanoma; however, the relevance and potential benefit of TBI as an immune modifying regimen has not been elucidated in detail thus far 21-24.

Hypothesizing that the modulating effects of low-dose TBI are ideal for successful Th1-based cancer immunotherapies, we investigated the impact of 2 Gy TBI on the host immune system and the migration properties of the transferred therapeutic antitumoral Th1 cells in healthy C3H and tumor-bearing RIP1-Tag2 mice. Furthermore, we identified resistance mechanisms, providing a rationale for our combined Th1-based immunotherapy approach.

## Methods

### Animals

C3HeB/FeJ (C3H) mice were purchased from the Jackson Laboratory (Bar Harbor, Maine, USA). Transgenic mice expressing a T cell receptor specific for Tag peptide 362-384 on CD4^+^ T cells (Tag2-TCR) and RIP1-Tag2 mice that develop pancreatic cancer under the control of the rat insulin promoter (RIP) were provided by Taconic and backcrossed to the C3HeB/FeJ strain.

Mice were bred in house under sterile and standardized environmental conditions with free access to food and water. For the *in vivo* studies, 8-12-week-old female C3H or 10 - 11-week-old female RIP1-Tag2 mice were used. All experiments were performed according to the animal use and care protocols of the German Animal Protection Law and approved by the local authorities (Regierungspräsidium Tübingen).

### Tag-Th1 cell culture

CD4^+^ T cells were isolated from the spleens and lymph nodes of Tag2-TCR donor mice using MACS CD4^+^ microbeads (L3T4; Miltenyi Biotec, Bergisch Gladbach, Germany). The detailed Th1 culture protocol was described previously 25, 26. Briefly, 2 × 10^5^ CD4^+^ T cells were cultured for 7 days with 5 × 10^5^ irradiated antigen presenting cells (APCs), Tag peptide 362-384 (20 μg/mL; EMC-microcollections, Tuebingen, Germany), CpG-DNA 1668 (0.2 μM; Eurofins Genomics, Ebersberg, Germany), IL-2 (5 U/mL; Novartis, Basel, Switzerland), and anti-IL-4 mAbs (10 μg/mL; from 11B11 hybridoma supernatant) to obtain a Tag-specific Th1 phenotype.

### 2 Gy total body irradiation (TBI)

TBI was applied in a single dose of 2 Gy using a caesium-137 gamma radiation source Gammacell© 1000 Elite unit (Nordion, Ontario, Canada). Mice were kept in groups of two in a specially designed cylindrical metal container for an irradiation time of 20 s, which equals a dose of 2 Gy. Control mice were sham-treated by placing the metal container with mice in the radiation unit without irradiation exposure.

### Combined immunotherapy (COMBO) of RIP1-Tag2 mice

RIP1-Tag2 mice (10 to 11 weeks old) were irradiated with 2 Gy 1 day prior to the first adoptive cell transfer with Tag-Th1 cells and every 4 weeks thereafter. Then, 10^7^ Tag-Th1 cells were transferred *intraperitoneally* (*i.p.*) weekly to mice. αPD-L1 and αLAG-3 mAbs treatment began the following day (500 µg each, *i.p.*) and was continued 1-2 times per week (200 µg). Blood glucose levels were measured twice a week as a valid marker for the tumor burden of insulin-producing islet cancer cells 27. If the blood glucose level declined below 30 mg/dL on two subsequent measurements, the animal was sacrificed according to the animal termination criteria.

### Splenectomy

Surgical removal of the spleen in one cohort of the mice was performed 1 week before therapy initiation under *i.p* anesthesia with medetomidine (0.5 µg/g body weight), midazolam (5 µg/g), and fentanyl (0.05 µg/g). The skin and peritoneum were opened by left-sided incision of the flank. The spleen was loosened, and supplying venules were tied before removal. Finally, the peritoneal wall and skin were closed by 2-3 sutures. Control groups underwent either sham surgery through a similar incision of the peritoneum or both sham surgery and sham treatment with isotype mAbs and NaCl instead of Tag-Th1 cells.

### Radiolabeling of ^64^Cu-DOTA-CD3 mAbs

The CD3 monoclonal antibody (mAb)-producing hybridoma cell line CRL-1975^TM^ was purchased from American Type Culture Collection (Manassas, Virginia, USA) and cultured in miniPERM Bioreactors (Sarstedt, Nümbrecht, Germany) with serum-free ISF medium (Biochrom, Berlin, Germany) at 37 °C and 5% CO_2_. 1,4,7,10-tetraazacyclododecane-1,4,7,10-tetraacetic acid (DOTA) conjugation and radiolabeling with copper-64 (^64^Cu) were performed as described previously 28. In short, diafiltered CD3 mAbs solution (8 mg/mL) was incubated with DOTA-NHS ester (10 mg/mL; Macrocyclics, Dallas, Texas, USA) for 24 h at 4 °C and adjusted to a concentration of 4-5 mg/mL. ^64^Cu was produced according to the protocol of McCarthy *et al.* 28, 29. Radiolabeling of the DOTA-conjugated CD3 mAbs with ^64^Cu was performed based on a slightly modified protocol from Lewis *et al.* 30. For this, 200 μg of DOTA-labeled CD3 mAbs were incubated with 100 MBq of ^64^Cu in 10 mM HCl at 40 °C for 40 min.

### CD3-ImmunoPET/CT

Mice were anesthetized with 1.5% isoflurane in 100% oxygen in a temperature-controlled anesthesia box. Approximately 11.1 MBq of the ^64^Cu-DOTA-CD3 mAbs were injected intravenously (*i.v.*) in TBI-treated or sham-treated C3H mice. Ten-minute static PET scans were performed 3, 24 and 48 h after tracer injection in a dedicated small-animal Inveon microPET scanner (Siemens Healthineers, Knoxville, Tennessee, USA). Reconstruction was applied by a statistical iterative ordered subset expectation maximization 2D algorithm (OSEM2D) without attenuation correction according to our standard mouse PET imaging protocol. To gain additional anatomical information, sequential CT scans were performed immediately after the PET scans on a dedicated small-animal combined high-resolution Inveon SPECT/CT scanner (Siemens) with an x-ray voltage of 70 keV and a current of 500 mA. Planar images were acquired in a 360° “step and shoot” mode with an exposure time of 350 ms, a binning factor of 4, and a pixel size of 75 μm and reconstructed to obtain 3-dimensional images.

After the final PET/CT scans, all animals were sacrificed by cervical dislocation under deep anesthesia. After dissection, organ weight was determined, and radioactivity was measured by γ-counting (Wallac 1480 WIZARD 3” Gamma Counter; PerkinElmer, Waltham, Massachusetts, USA) using an energy window between 350 and 650 keV. For quantification, a standardized aliquot of the injected radiotracer was added to the measurement. Acquired PET-images were corrected for radioactive decay, normalized to the injected activity, and finally coregistered to the CT scan using Inveon Research Workplace (Siemens). The biodistribution results for each organ are expressed as the percentage of the overall injected dose per g (%ID/g) or injected dose per organ (%ID).

### Optical Imaging

For optical imaging (OI) studies, 2 ×10^6^ Tag-Th1 cells/mL were labeled with 5 µl/mL fluorescent Vybrant DiD cell-labeling solution (Thermo Fischer Scientific, Waltham, Massachusetts, USA) for 2 min on a roll mixer and washed twice with PBS immediately before adoptive cell transfer. Then, 10^7^ Tag-Th1 cells were injected *i.p.* per mouse.

Before *in vivo* cell tracking analysis, mice were shaved and depilated at the trunk. OI acquisitions were performed under 1.5% isoflurane/oxygen anesthesia with a Aequoria OI system (Hamamatsu Photonics, Hamamatsu, Japan) containing an ORCA-II-ER charge-coupled device camera. We acquired 2-dimensional bright field images followed by fluorescence acquisitions with standardized parameters. DiD was excited with a 650±20 nm bandpass filter at 100% light intensity, and the DiD-emission was detected after 20 s of exposure and closed aperture via a 690±30 nm blocking filter. Afterwards, the animals were sacrificed and dissected for *ex vivo* OI scans of the organs to reveal the exact homing sites of the Tag-Th1 cells. The image analysis was performed using the Wasabi software (Hamamatsu). *Ex vivo* organ signal intensities were quantified by drawing region of interests (ROIs) around each organ and divided by the signal intensities of correspondingly sized “black” background ROIs.

### Flow cytometry

Single-cell suspensions were achieved by passing the lymphatic organs through 40 µM cell strainer sieves. Blood was taken retrobulbary under anesthesia and collected in EDTA microvettes (Sarstedt, Nümbrecht, Germany). To determine quantitative cell numbers, cells were counted on a Neubauer counting chamber (Assistant, Sondheim, Germany) using Trypan blue. The following fluorescent dyes purchased from BD (Franklin Lakes, New Jersey, USA) were used for staining in the C3H experiments: V500-CD45 (30-F11), APC/Cy7-CD3ε (500A2), PE/Cy7-CD4 (GK1.5), FITC-CD8a (53-6.7), APC/Cy7-CD19 (ID3), PE/Cy7-CD11b (M1/70), FITC-CD11c (HL3), PE-CD49b (DX5), PE-FoxP3 (MF23), PerCP/Cy5.5-CD25 (PC61), PerCP/Cy5.5-Gr-1 (RB6-8C5), PB-CD4 (GK1.5), and B220 (RA3-6B2). FVS450 served as a viability stain. For the RIP1-Tag2 experiments, the following dyes were purchased from BioLegend (San Diego, California, USA): AF700-CD45 (30-F11), AF700-CD8a (53-6.7), APC-LAG-3 (C9B7W), APC-CD11c (N418), BV421-CD25 (PC61), BV421-CD3ε (145-2C11), BV510-CD11b (M1/70), BV510-CD44 (IM7), BV605-Tim-3 (RMT3-23), BV605-PD-L1 (10F.9G2), BV650-CD69 (H1.2F3), BV711-CD115 (AFS98), BV711-PD-1 (29F.1A12), BV785-CD127 (A7R34), BV785-B220 (RA3-6B2), PE-NKp46 (29A1.4), PE/Dazzle594-CTLA-4 (UC10-4B9), PE/Dazzle594-F4/80 (BM8), PE-Cy7-Ly6C (HK1.4), PE/Cy7-CD62L (MEL-14), FITC-Ly6G (1A8), FITC-CD3ε (500A2), PerCP-I-A/I-E (M5/114.15.2), PerCP-CD4 (GK1.5) and Zombie NIR Fixable Viability Dye. Tag-TCR targeting 9H5.1 mAbs were produced in house from hybridoma supernatant and PE-labeled using a Lightning-Link® Conjugation Kit (Innova Biosciences, Cambridge, UK) according to the manufacturer's protocol. Fcγ receptors were blocked with CD16/32 mAb to minimize nonspecific labeling. For intracellular staining, cells were resuspended in BD Cytofix/Cytoperm Buffer^TM^ prior to staining with FITC-FoxP3 (259D/C7).

Cell suspensions were measured on a BD LSR-II or LSRFortessa cytometer (both BD). Single stains, minus-one stains, isotype controls, and/or compensation beads (BD) were used as controls and for compensation of spillover in multicolor-analyses. The data were analyzed using FlowJo 10 (FlowJo, Ashland, Oregan, USA). The gating strategies of both panels are illustrated in Figure S1. The percentage of viable CD45^+^ cells for each cell population was stated as relative cell numbers, whereas absolute cell numbers were calculated by relative cell number × total viable white blood cell count of the respective organ.

### Cytokine assay

Blood from mice was collected retrulbary under anesthesia. After centrifugation and separation from cells, sera were diluted 1:1 with PBS and stored at -80 °C until shipping. Samples (Duplicates of each mouse) were analyzed by Eve Technologies (Calgary, Canada) with a 32-Plex BioPlex 200 Mouse Cytokine/Chemokine Array.

### Statistical analyses

The data were analyzed using GraphPad Prism, Version 7 (GraphPad Software, Inc., San Diego, California, USA). Values are expressed as the arithmetic means ± standard deviation (SD) if not otherwise indicated. For statistical analyses, unpaired t-tests were applied corrected for multiple comparisons using the Holm-Sidak-method. For multiple group comparisons, one-way ANOVA was performed with Holm-Sidak as a post hoc test. Survival curves were analyzed with the log-rank-test. A value of P < 0.05 was considered statistically significant (*).

## Results

### Efficacy of combined Th1 cell and dual ICB therapy crucially depend on 2 Gy TBI

**Figure 1:**
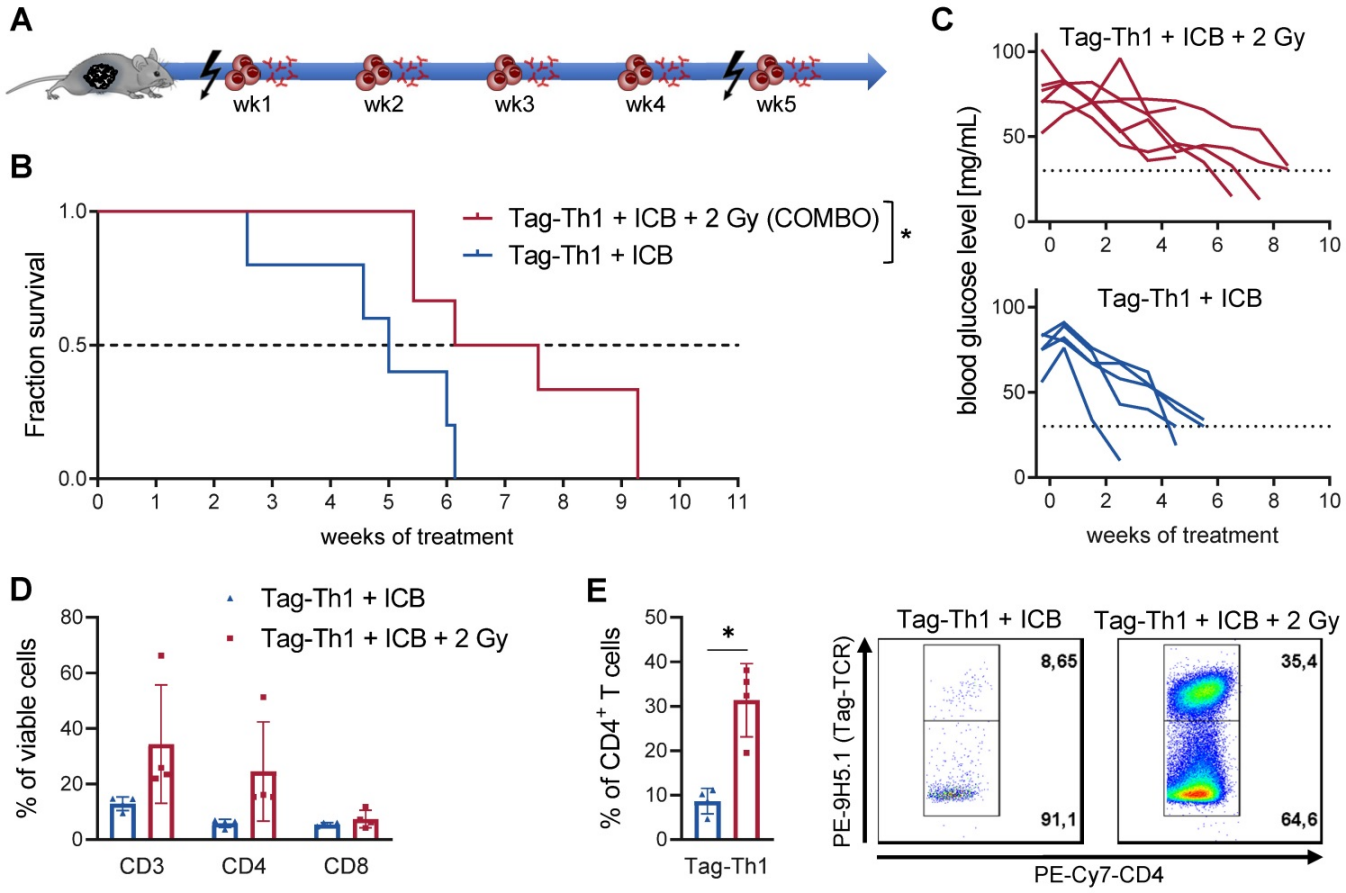
** Combined immunotherapy of Tag-Th1 cells and dual immune checkpoint blockade (ICB) is crucially dependent on 2 Gy TBI.** (**A**) RIP1-Tag2 mice at 10-11 weeks of age received weekly adoptive transfers of 10^7^ Tag-Th1 cells followed by PD-L1/LAG-3 blocking mAbs 24 h later. One day prior 1^st^, 5^th^, and 9^th^ cell application mice were 2 Gy TBI or sham irradiated. (**B**) Survival of RIP1-Tag2 mice treated with combined immunotherapy of Tag-Th1 cells, αPD-L1/αLAG-3 mAbs with (COMBO, red) or without 2 Gy TBI (blue) (n = 5-6 per group). (**C**) Blood glucose levels, representing a reliable blood marker for tumor burden of insulin-producing islet carcinoma cells, confirm rapid tumor progression in the non-irradiated treatment group (blue) compared to the COMBO group (red). (**D**) Another cohort of mice (n = 4 per group) was sacrified 10 days after first Tag-Th1 cell administration and immune cell infiltrates of the pancreatic tumor tissue were analyzed by flow cytometry. Irradiation impacted the fractions of CD3^+^ T cells (CD3) and CD3^+^CD4^+^ T cells (CD4) and to a lesser extend CD3^+^CD8^+^ T cells (CD8). (**E**) The ratio of Tag-Th1 cells to all CD4^+^ cells increased when 2 Gy TBI was added to the treatment.

The combined therapy (COMBO) of 2 Gy TBI + weekly adoptive transfer of 10^6^ Tag-Th1 cells and dual PD-L1/LAG-3 immune checkpoint blockade significantly prolonged the survival of RIP1-Tag2 mice with late-stage metastasized pancreatic islet carcinomas 8, 9. To explore the impact and immune modulatory effects of 2 Gy TBI, we compared the COMBO treatment efficacy with and without irradiation of RIP1-Tag2 mice (Figure 1A, therapy regimen). Overall survival of COMBO treated mice was markedly diminished, when 2 Gy was not applied as preparative regimen (Figure 1B). In line with this, blood glucose levels, a reliable marker for insulin-producing tumor cell burden, was stable over the first treatment weeks, but continuously decreased in non-irradiated RIP1-Tag2 mice (Figure 1C). Another cohort of mice received the similar treatment protocol and was sacrificed 10 days after Tag-Th1 cell administration to analyze immune cell infiltration of the pancreatic tumor tissues by multicolor flow cytometry. CD3^+^ and CD4^+^ T cell infiltrates increased by 2.5- and 4-fold (non-significant), respectively, but not CTL when 2 Gy was applied to the Tag-Th1 + PD-L1/Lag-2 treatment (Figure 1D). Notably, in the pancreata of 2 Gy TBI treated mice, 31.4±8.3% of the CD4^+^ cells were adoptive Tag-Th1 cells compared to 8.6±2.9 in the non-irradiated therapy group (Figure 1E).

### 2 Gy TBI induces moderate but sustainable lymphocyte depletion in blood and lymphatic organs

To gain a deeper understanding in the underlying mechanisms, enabling the treatment efficacy, we started to study the immunologic effects of 2 Gy TBI in healthy mice without immunotherapeutic intervention. C3H mice, representing the background strain of the RIP1-Tag2 mouse model, were used to investigate immunological alterations after TBI. All mice tolerated 2 Gy TBI without showing any clinical signs of agitation, weight loss or reduced food intake for a maximal observation period of 28 days.

**Figure 2:**
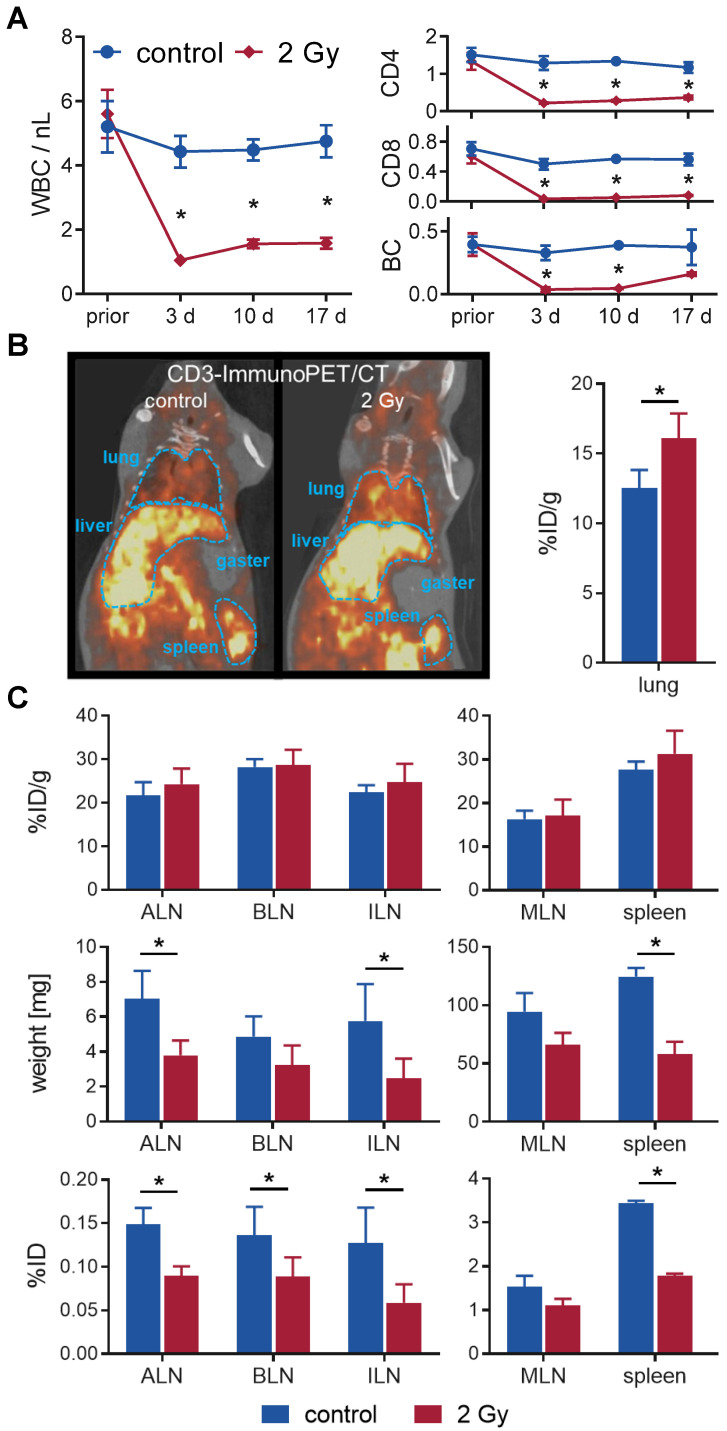
** TBI-induced moderate lymphodepletion of the host.** (**A**) Repetitive white blood cell (WBC) count (left) and corresponding flow cytometric analyses of lymphocyte populations (right) of 2 Gy TBI-treated (♦) and sham-treated (●) C3H mice over 17 days (n=5-8 per group). (**B**) Representative *in vivo* CD3-ImmunoPET/CT images (left) and *ex vivo* organ quantification by γ-counting (right) 48 h after *i.v.* administration of ^64^Cu-DOTA-CD3 mAb (72 h post-TBI) revealed higher irradiation-induced uptake in the lung (n = 3 per group). Mean percent injected dose per gram, %ID/g. (**C**) Weight- and decay-adjusted organ biodistribution (%ID/g, above) of the lymphatic organs were similar between the experimental groups. Organ weight reduction after TBI (middle) provoked significant decreases in the absolute uptake per organ (%ID, below) in TBI-treated mice. ALN = axillary lymph nodes, BLN = brachial lymph nodes, ILN = inguinal lymph nodes, MLN = mesenteric lymph nodes.

In a longitudinal setting, blood was taken repetitively from mice with or without TBI over 17 days to determine the impact on white blood cell (WBC) count and lymphocyte populations (Figure 2A). WBC significantly decreased from 5.6±0.7/nL to 1.0±0.1/nL after 3 days and only slightly reconstituted by day 17 (1.6±0.2/nL vs. 4.8±0.5/nL in sham-treated control mice). Flow cytometric analyses of the lymphocyte populations 3 days after TBI revealed that B cells (TBI: 0.04±0.02/nL, control: 0.33±0.06/nL) and CD8^+^ T cells (0.03±0.01/nL vs. 0.50±0.07/nL) were more affected by TBI than CD4^+^ T cells (0.2±0.0/nL vs. 1.3±0.2/nL). In contrast, B cells (0.16±0.01/nL, 4.4-fold) and CD8^+^ T cells (0.08±0.01/nL, 2.3-fold) recovered to a larger extent by day 17 compared to CD4^+^ T cells (0.36±0.05/nL, 1.7-fold) (Figure 2A).

To investigate irradiation-induced changes in lymphocyte distribution *in vivo* by PET/CT, we injected T cell targeting ^64^Cu-DOTA-CD3 mAb 24 h after 2 Gy TBI or sham treatment into C3H mice. PET/CT studies exhibited a pronounced T cell accumulation in the lungs of irradiated mice 48 h after ^64^Cu-DOTA-CD3 mAb injection (72 h post-TBI), confirmed by *ex vivo* biodistribution analysis (16.1±1.8 %ID/g, TBI vs. 12.5±1.3 %ID/g, control) (Figure 2B). Furthermore, we focused on ^64^Cu-DOTA-CD3 mAb biodistribution, measured by *ex vivo* γ-counting, within the secondary lymphatic organs of C3H mice (Figure 2C) but could not observe any differences in the weight adjusted tracer uptake (%ID/g) in the spleen and lymph nodes. Most importantly, the weight of the spleens, axillary and inguinal lymph nodes decreased to approximately 50% by day 3 after TBI (mean spleen weight, 2 Gy TBI: 58±11 mg; control: 125±7 mg), causing significantly lower total ^64^Cu-DOTA-CD3 mAb uptake (%ID) in the secondary lymphatic organs if not corrected for weight. As researchers generally refer to relative cell concentrations, this considerable TBI-induced loss of organ volume in mice but not T cell density is of paramount importance for the interpretation of T cell distribution studies related to TBI and other lymphodepleting regimens.

### TBI enhances adoptive Tag-Th1 migration into the liver, lung and lymph nodes in C3H mice

**Figure 3:**
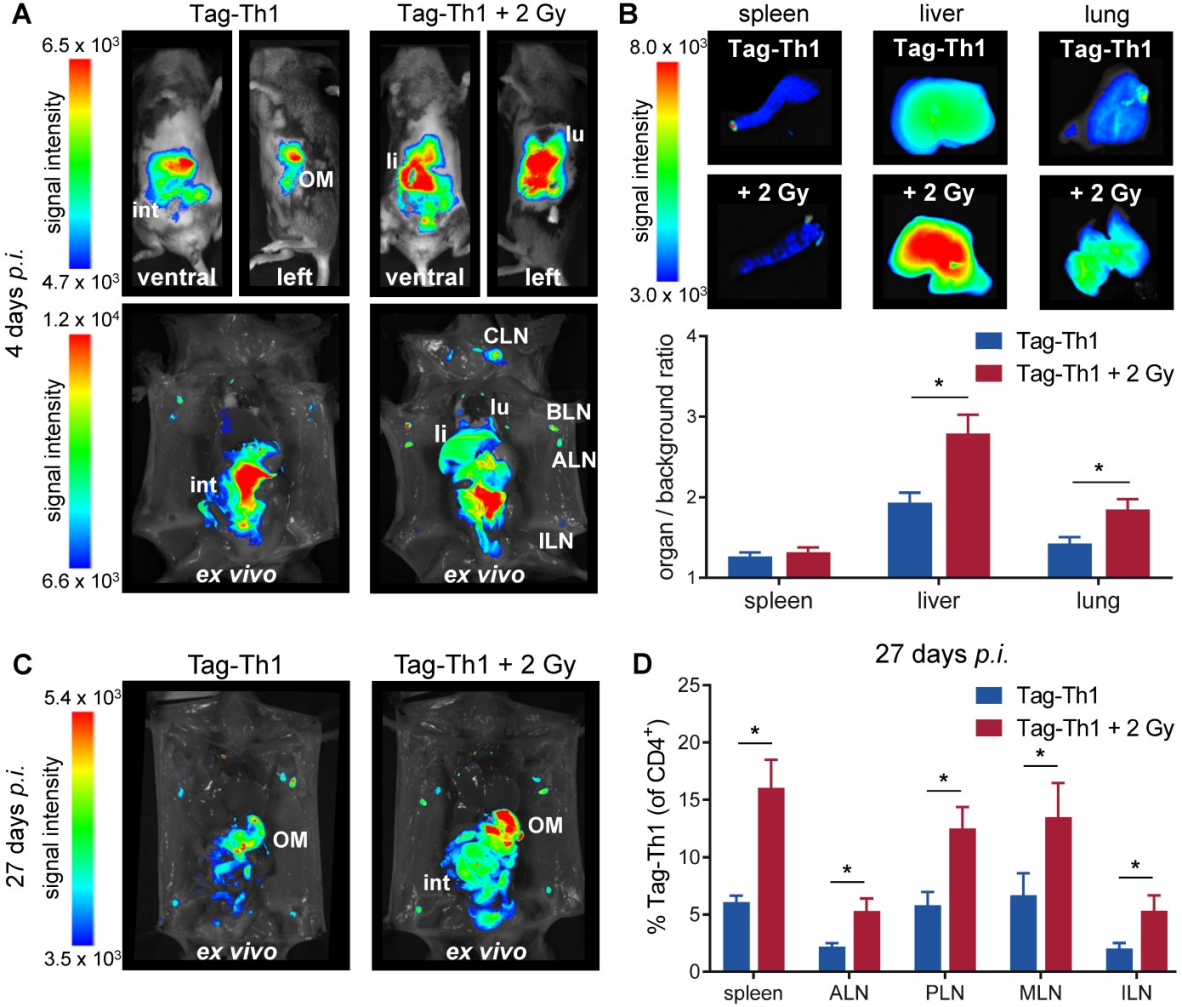
** Adoptively transferred Tag-Th1 cells preferentially migrate to liver, lung, spleen and lymph nodes after TBI.** (**A**) Representative *in vivo* (top) and *post-mortem* (lower) optical images of TBI-treated and sham-treated C3H mice 4 days after application of 10^7^ DID fluorescently-labeled Tag-Th1 cells (n = 5 per group). 2 Gy TBI was performed 1 day prior cell administration. (**B**) Organ biodistribution quantified by the organ-to-background ratio revealed higher Tag-Th1 cell derived fluorescence signals in the liver and lung after 2 Gy TBI. (**C**) *Post-mortem* optical imaging of representative TBI-treated and untreated C3H mice 27 days after administration of 10^7^ DID fluorescently-labeled Tag-Th1 cells (28 days post-2 Gy TBI) exhibited long term accumulation in the peritoneum, primarily the omentum majus (OM) and lymph nodes (n = 5 per group). (**D**) Tag-Th1 fractions of the entire CD4^+^ T cell populations in the spleen and lymph nodes after TBI (red) compared to the control group (blue) as analyzed by flow cytometry. lu = lung, li = liver, int = intestinum, cervical (CLN), axillary (ALN), brachial (BLN), inguinal (ILN) lymph nodes.

To study whether TBI alters the migration properties of adoptively transferred CD4^+^ T cells in a non tumor-bearing host, we applied 10^7^ DID-fluorescently labeled Tag-Th1 cells *i.p.* to C3H mice 24 h after TBI in accordance with our established T cell-based immunotherapy of RIP1-Tag2 mice 6. OI studies were performed at 4, 10, and 27 days after *i.p.* injection of Tag-Th1 cells to follow cell distribution dynamics. Whole-body OI investigations revealed a pronounced Tag-Th1 cell-derived signal within the liver and lung as a consequence of 2 Gy TBI 4 days (Figure 3A and S2A) and 10 days (Figure S2B) post-injection. These results were confirmed by quantitative *ex vivo* OI analyses. At day 4, the organ-to-background-ratio was significantly higher in the livers (2.8±0.2, TBI vs. 1.9±0.1, control) and lungs (1.8±0.1 vs. 1.4±0.1) of TBI-treated mice, whereas no significant difference was determined in the spleens (Figure 3B and S2B). To confirm stable DiD labeling of adoptive Tag-Th1 cells without phagocytic DiD incorporation, we further conducted flow cytometric analysis employing a Tag-T cell receptor-specific mAb (9H5.1, Figure S2C). Twenty-seven days after administration, Tag-Th1 cells mainly accumulated in the lymph nodes and peritoneal cavity, indicating long term persistence of the transferred Tag-Th1 cells (Figure 3C). In addition, we isolated spleens, mesenteric and extraperitoneal lymph nodes for flow cytometric analysis at day 27. The Tag-Th1 cell fraction in these lymphatic organs was significantly higher in TBI mice at this late time point, proving their reinforced homing through TBI (Figure 3D).

### TBI reconfigures the immune cell composition and strongly increases Tag-Th1 cell ratios in blood and lymphatic organs

**Figure 4:**
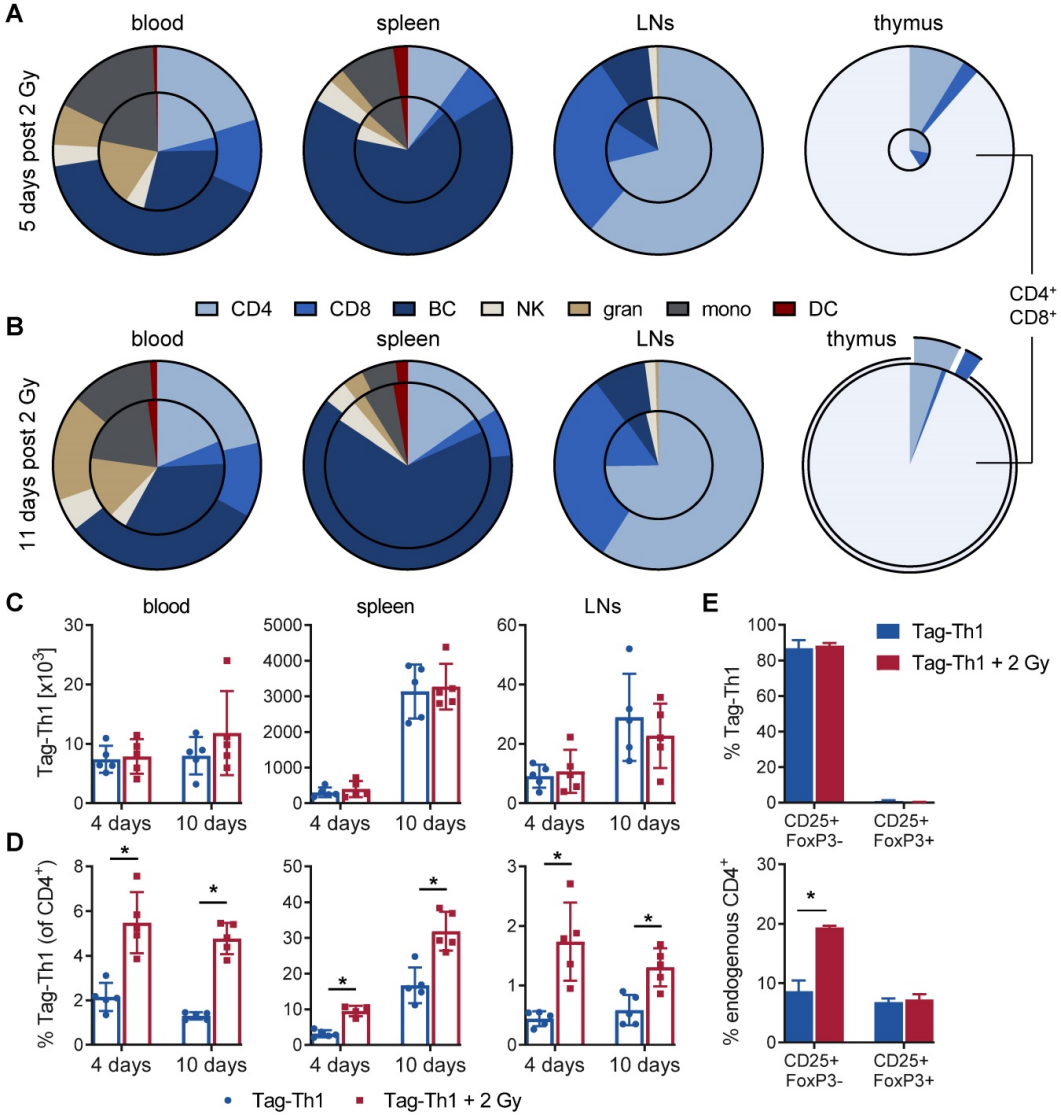
** Reconfiguration of the host immune cell composition by 2 Gy TBI results in increased Tag-Th1 cell fractions.** Flow cytometric analyses (**A**) 5 and (**B**) 11 days post-2 Gy TBI of the main immune cell populations in the blood, spleen, extraperitoneal lymph nodes (LN) and thymus (n = 5 per group). The decrease of viable CD45^+^ cells after TBI is delineated by the area of the inner circle in comparison to the outer circle (non-irradiated control cohort). Within each circle the relative fraction of each cell population is color-coded. CD4 = CD3^+^CD4^+^ T cells, CD3 = CD3^+^CD8^+^ T cells, BC = CD19^+^ B cells, NK = CD49b^+^ natural killer cells, gran = CD11b^+^Gr-1^High^ granulocytes, mono = CD11b^+^Gr-1^low^ monocytes/macrophages, DC = CD11c^+^ dendritic cells. (**C**) Absolute cell numbers of TBI-treated (red) and untreated C3H mice (blue) 4 and 10 days after Tag-Th1 cell administration in blood (cells/γL), spleen, and extraperitoneal lymph nodes as analyzed by flow cytometry. Absolute cell numbers were calculated by WBC count of each organ x Tag-Th1 cell fraction of viable CD45^+^ cells. (**D**) Relative Tag-Th1 cell fractions of total CD4^+^ T cells after TBI in all analyzed organs. (**E**) Fractions of functionally active (CD25^+^FoxP3^-^) and regulatory (CD25^+^FoxP3^+^) transferred Tag-Th1 cells and endogenous CD4^+^ T cells in the lymph nodes 4 days after cell application.

Subsequently, we analyzed the impact of 2 Gy TBI on the host immune cell composition and Tag-Th1 cell ratios in blood, spleen lymph nodes, and thymus by multicolor flow cytometry at the earlier time points. 5 days after TBI, the absolute number of WBC decreased to 32% in the blood (in relation to nonirradiated controls) and to 26% in the spleens and extraperitoneal lymph nodes (Figure 4A and S3). In accordance with our longitudinal study (Figure 2A), endogenous CD4^+^ T cells exhibited less irradiation-induced depletion in the blood (35%), spleen (26%), and lymph nodes (29%) compared to CD8^+^ T cells (blood: 11%; spleen: 9%; lymph nodes: 11%). The B cell depletion in the blood (26%), spleen (25%), and lymph nodes (40%, not significant) was comparable to the depletion of CD4^+^ T cells. As early as 11 days after 2 Gy TBI, we already observed a partial WBC reconstitution in the blood (42% of nonirradiated control) and spleen (62%) with largely increased CD4^+^/CD8^+^ cell ratios (Figure 4B and S3). Granulocytic and monocytic cells accounted for a larger proportion of WBCs at both time points, thus being less affected by 2 Gy TBI than lymphoid cells (Figure 4A-B, and S3).

As the thymus is crucial for T cell development and differentiation, we also investigated the impact of 2 Gy TBI on thymic cells. Five days after TBI, the thymic WBC count decreased dramatically to 4.9% (Figure 4A and S3). Thus, 2 Gy thymic cell depletion was by far the highest of all analyzed lymphatic organs, but complete reconstitution appeared already on day 11, which is in line with slightly higher immune cell counts in the blood and spleen at this time point (Figure 4B and S3). Interestingly, double-positive CD4^+^CD8^+^ (undifferentiated) thymic T cells, representing the vast majority of thymic cells, and CD4^+^ T cells almost recovered to the initial cell number by day 11 post-TBI but not CD8^+^ T cells. Moreover, CD3 expression by thymic T cells, a marker for T cell differentiation, increased from 7% at day 5 to 73% at day 11 post-TBI, confirming the rapid reconstitution of lymphocytes in the thymus (Figure S3).

Next, we analyzed the Tag-Th1 cell organ distribution and Tag-Th1 cell ratios to other immune cell populations. In TBI and control mice, the absolute Tag-Th1 cell numbers in the blood, spleens or extraperitoneal lymph nodes were similar 4 days and 10 days after adoptive Tag-Th1 cell transfer (day 5 and day 10 post-TBI) (Figure 4C). Tag-Th1 cells accumulated in the spleen irrespective of 2 Gy TBI (TBI: 3.3±0.6 x 10^6^; control: 3.1±0.8 x 10^6^) on day 10.

However, 2 Gy TBI-induced lymphodepletion yielded significant increased ratios of Tag-Th1 cells to host CD4^+^ T cells in all analyzed organs of interest, ranging from 1.9-fold in the spleen to a 4-fold higher ratio in lymph nodes (Figure 4D). Additionally, over 80% of the adoptively transferred Tag-Th1 cells stained positive for the activation marker CD25 but negative for the regulator T cell marker FoxP3 in the lymph nodes 4 days after *i.p.* administration (Figure 4E). Of note, lymph nodes of 2 Gy TBI mice exhibited a significantly higher fraction of CD25^+^FoxP3^-^ endogenous CD4^+^ T cells compared to nonirradiated C3H mice (Figure 4E).

Based on these findings, we calculated the Tag-Th1 cell ratios to all other analyzed immune cell populations for the early (day 4; Table S1) and late (day 10; Table S1) time points. At day 4 post-cell administration, the Tag-Th1 cell to CD8^+^ T cell ratio increased greater 10-fold, the Tag-Th1 cell to B cell ratio 3- to 7-fold and the Tag-Th1 cell to NK cell ratio 2- to 3-fold in the blood, spleen, and lymph nodes by 2 Gy TBI. Increased Tag-Th1 cells to immune cell ratios persisted 10 days after Tag-Th1 cell administration. Generally, at day 4 and 10, the Tag-Th1 cell to granulocyte and monocyte ratios increased but were less pronounced than the Tag-Th1 cell to lymphoid cell ratios.

### TBI fosters host and adoptive CD4^+^ T cell accumulation in tumor-bearing pancreata

**Figure 5:**
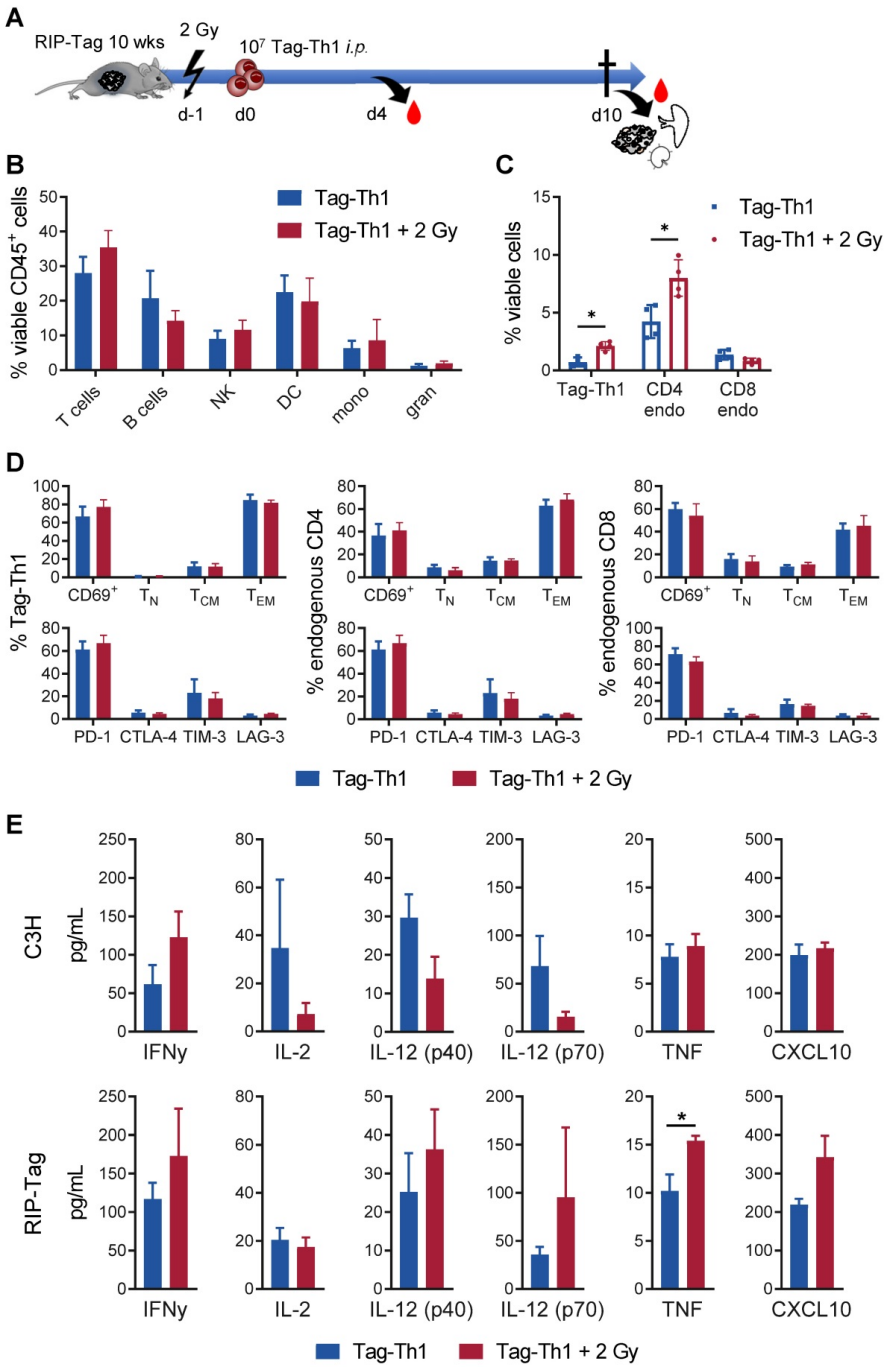
** Low-dose TBI provokes enhanced tumor-infiltration of Tag-Th1 and endogenous CD4^+^ T cells in RIP1-Tag2 mice.** (**A**) 10^7^ tumor antigen-specific Tag-Th1 cells were injected *i.p.* into 10- to 11-week-old RIP1-Tag2 tumor-bearing mice with progressed tumors 1 day after 2 Gy TBI or sham-treatment. Pancreas, spleen, and pancreas draining lymph nodes were harvested for *ex vivo* analyses 10 days after cell administration (11 days after 2 Gy TBI) (n = 4 per group). (**B**) Immune cell infiltrates of pancreatic tumor tissue of nonirradiated (blue) and 2 Gy-irradiated (red) mice were analyzed by multicolor flow cytometry. Cell subsets were classified as CD3^+^ T cells, CD19^+^ B cells, NKp46^+^ NK cells (NK), CD11c^+^ dendritic cells (DC), CD11b^+^Ly6G^-^ macrophages/monocytes (mono), and CD11b^+^Ly6G^+^ granulocytes. (**C**) Flow cytometric analyses revealed higher infiltrates of adoptive Tag-Th1 cells, endogenous CD3^+^CD4^+^ T cells (CD4 endo), but not endogenous CD3^+^CD8^+^ T cells (CD8 endo) cells. (**D**) Adoptively transferred and host T cells isolated from the spleen were analyzed for activation status (CD69^+^), phenotypic differentiation (CD44^-^CD62L^+^ naïve (T_N_), CD44^+^CD62L^+^ central memory (T_CM_), CD44L^+^CD62L^-^ effector memory (T_EM_) T cells) and expression of checkpoint molecules. (**E**) Blood levels of Th1-associated cytokines (mean±SEM) of healthy C3H and tumor-bearing RIP1-Tag2 mice 4 days after Tag-Th1 cell administration and 5 days after 2 Gy TBI (or sham-irradation) (n = 4-5 per group). The values of each individual mouse were derived from the arithmetic mean of duplicates.

Next, we studied the immunological effects of 2 Gy TBI on the endogenous RIP1-Tag2 pancreatic islet tumor mouse model, in which every islet cell expresses the Tag2-antigen. In this setting, 10 - 11-week-old female RIP1-Tag2 mice underwent 2 Gy TBI and were *i.p.* injected with 10^7^ Tag-Th1 cells 24 h later (Figure 5A). 10 days after Tag-Th1 cell administration (11 days after 2 Gy TBI), the insular cell carcinoma-bearing pancreata, the pancreatic draining lymph nodes (PLN), and spleens were isolated for multicolor flow cytometry.

In the pancreatic tumor tissue, the T cell fraction slightly increased as a consequence of 2 Gy TBI (TBI: 35.4%; control: 28.0%, not significant) (Figure 5B). No relevant changes were observed in the other immune cell populations.

In line with previous experiments 31, we detected only very few F4/80^+^ tumor-associated macrophages (2.39% of viable CD45^+^ cells, both groups) after Tag-Th1 cell transfer in non-irradiated and TBI treated mice (Figure S4A). 67.9% (non-irradiated) and 78.6% (2 Gy TBI) of the macrophages derived from pancreatic carcinomas stained positive for the M1-marker MHC-II, suggesting minor impact of 2 Gy TBI on tumor-suppressive macrophages in this therapy model. Notably, 2 Gy TBI elevated the fraction of adoptively transferred Tag-Th1 cells by three-fold in the pancreatic tissue compared to nonirradiated RIP1-Tag2 mice (Figure 5C). Similarly, the number of endogenous CD4^+^ T cells increased by two-fold after 2 Gy TBI, whereas the number of endogenous CD8^+^ T cells remained unaffected. In sharp contrast, the fraction of endogenous CD4^+^ and CD8^+^ T cells decreased in the PLNs as a consequence of 2 Gy TBI, whereas the fraction of adoptively transferred Tag-Th1 cells remained stable (Figure S4B-C).

The majority of Tag-Th1 cells in the tumor (Figure 5C) and PLN (Figure S4D) expressed the lymphocyte activation-associated membranous antigen CD69 independent of TBI, were further classified as effector memory T cells (CD44^+^CD62L^-^) and stained positive for the immune checkpoint PD-1. This phenotype applies in the same manner to the endogenous CD4^+^ and CD8^+^ T cells within the tumor but not the PLNs. In the PLNs, most endogenous host T cells expressed a naïve (CD44^-^CD62L^+^) phenotype (Figure S4D).

### TBI induces a systemic tumor-specific Th1 immune response but no unintended cytokine release

Furthermore, we analyzed protein expression of the Th1-specific cytokines TNF, IFN-γ, IL-2, IL-12 (p40), IL12 (p70) and the IFNγ-driven chemokine CXCL10 in the sera of RIP1-Tag2 and C3H mice 4 days after adoptive Tag-Th1 cell transfer (5 days post-2 Gy TBI) to uncover whether 2 Gy TBI fosters Th1-mediated immune responses systemically. In C3H mice, 2 Gy TBI yielded considerable (nonsignificant) increases in IFNγ secretion but not TNF or CXCL10. Surprisingly, IL-2 and IL12 protein expression decreased following 2 Gy TBI (Figure 5E). In sharp contrast, the analysis of Th1-dedicated cytokines in the sera derived from tumor antigen-expressing RIP1-Tag2 mice 5 days after TBI exhibited a significant (~30%) increase in TNF and non-statistically significant but apparent tendencies towards enhanced IFN-γ, IL-12, and CXCL10 protein release compared to RIP1-Tag2 mice without TBI, indicating promotion of a Tag2-specific antitumoral Th1 immune response (Figure 5E). Cytokine levels of the complete 32-plex panel revealed no TBI-induced upregulation of the Th2-associated cytokines IL-4 (<1 pg/mL), IL-5 or IL-13 but increased T helper 9 (IL-9) and T helper 17 (IL-17) cell protein levels in the sera of RIP1-Tag2 mice 4 days after Tag-Th1 cell transfer (Figure S5). Furthermore, the monocyte attractant protein CCL-2, which is associated with protumor activity, and granulocyte colony stimulating factor (G-CSF), which is essential for the maturation and differentiation of neutrophils, decreased by more than two-fold following 2 Gy TBI. In general, we observed no considerable increase in cytokine release 5 days (Figure S5) or 11 days (data not shown) after 2 Gy TBI. Thus, there was no evidence of an exaggerated unintended systemic immune activation when adoptive Th1 cell therapy was combined with 2 Gy TBI.

### TBI-mediated Th1 immune responses are negatively regulated by the spleen

**Figure 6:**
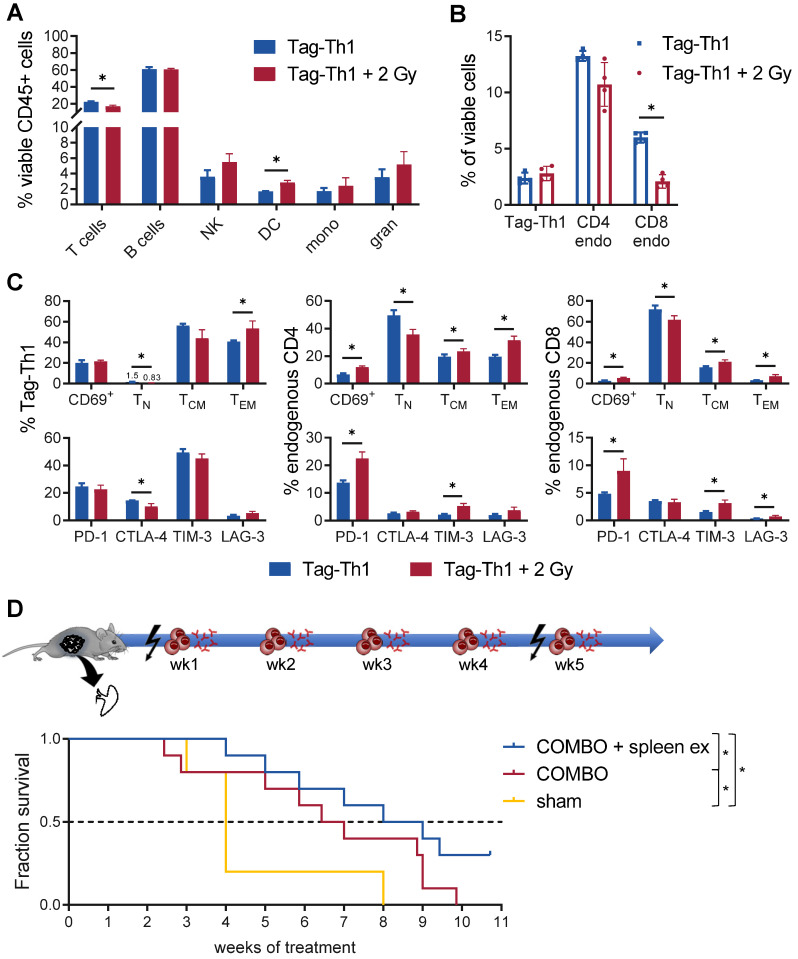
** Th1-based immunotherapy is negatively regulated by the spleen.** (**A**) Immune cell infiltrates of the spleen of nonirradiated (blue) and 2 Gy TBI (red) mice analyzed 10 days after Tag-Th1 cell administration (11 days post-2 Gy TBI) by multicolor flow cytometry (n = 4 per group). Cell subsets were classified as CD3^+^ T cells, CD19^+^ B cells, NKp46^+^ NK cells (NK), CD11c^+^ dendritic cells (DC), CD11b^+^Ly6G^-^ macrophages/monocytes (mono), and CD11b^+^Ly6G^+^ granulocytes. (**B**) Splenic T cell fractions of Tag-Th1 cells, endogenous CD3^+^CD4^+^ (CD4 endo) and CD3^+^CD8^+^ T cells (CD8 endo). (**C**) Adoptively transferred and host T cells isolated from the spleen were analyzed for activation status (CD69^+^), phenotypic differentiation (CD44^-^CD62L^+^ naïve (T_N_), CD44^+^CD62L^+^ central memory (T_CM_), CD44L^+^CD62L^-^ effector memory (T_EM_) T cells) and expression of checkpoint molecules. (**D**) RIP1-Tag2 mice at 10-11 weeks of age received weekly adoptive transfers of 10^7^ Tag-Th1 cells followed by PD-L1/LAG-3 blocking mAbs 24 h later. One day prior, 1^st^ and 5^th^ cell application mice were 2 Gy TBI or sham treated. Spleens of RIP1-Tag2 mice were surgically removed in one experimental group one week prior to therapy initiation (COMBO + spleen ex). The other groups underwent either sham surgery and COMBO treatment (COMBO) or both sham surgery and sham treatment (sham) (Treatment groups: n = 10, sham group: n = 5).

Furthermore, we observed a significant impact of TBI on the immune cell composition of the spleen, as endogenous CD4^+^ (not significant) and CD8^+^ T cells diminished (Figure 6A-B). In contrast, not the total fraction but the effector memory phenotype of adoptively transferred Tag-Th1 cells increased after 2 Gy TBI (Figure 6C). Similarly, the naïve subset of endogenous CD4^+^ and CD8^+^ T cells decreased in favor of antigen-experienced effector memory T cells (Figure 6C). In addition, 2 Gy TBI promoted the activation of splenic T cells, as we determined increased expression of the activation marker CD69 by endogenous CD4^+^ and CD8^+^ T cells when compared to T cells derived from littermates without TBI (Figure 6C). Interestingly, 2 Gy TBI also elevated the expression patterns of the immune checkpoints PD-1, TIM-3 and LAG-3 by endogenous T cells but not the therapeutic Tag-Th1 cells. CTLA-4 expression was even downregulated in splenic Tag-Th1 cells of TBI-treated mice.

Based on the above findings, we concluded that preparative TBI modulates the immune system towards a Th1-directed immune response, but treatment effects might be limited due to the induction of T cell exhaustion, mainly in the spleen. This observation could further explain the therapeutic efficacy of the COMBO treatment with 2 Gy + Tag-Th1 + dual PD-L1/Lag-3 immune checkpoint blockade in late-stage RIP1-Tag2 mice with metastatic insular carcinomas, while treatment with 2 Gy + Tag-Th1 cells alone was not efficient 8. As the spleen was heavily affected by 2 Gy TBI, and we demonstrated previously that the spleen is one of the first homing sites of *i.p.* adoptively transferred Tag-Th1 cells 31, we studied the impact of splenectomy on the therapeutic efficacy of our COMBO approach. Thus, we surgically removed the spleens of RIP1-Tag2 mice at 9-10 weeks of age and initiated COMBO 1 week later when mice recovered from the surgery and exhibited already progressed insular cell carcinomas as determined by decreased blood glucose levels. Experimental COMBO-treated RIP1-Tag2 mice that underwent splenectomy survived significantly longer compared to sham-operated but COMBO-treated or sham-operated and sham-treated control mice (Figure 6D). These results supported our findings that the spleen negatively regulates Th1-based cancer immunotherapy, likely by inducing T cell exhaustion that can be effectively diminished by combining Tag-Th1 cells with dual immune checkpoint blockade and 2 Gy TBI.

## Discussion

In recent years, considerable advances in the treatment of metastatic cancer have been achieved mainly by employing ICB and CAR T cell therapies in clinical practice. Given the fact that the majority of patient acquire primary or secondary resistance to immunotherapies, it is crucial to develop rational treatment combinations 32, 33. Recent data indicate the need of favorable cytokine profile for effective ACT, rather than extensive immune suppression 34, 35. Here, we demonstrated that a low-dose TBI of 2 Gy was sufficient to achieve a superior treatment efficacy employing tumor-directed Th1 cells and dual ICB to RIP1-Tag2 mice with progressed pancreatic islet carcinomas.

In our experiments, 2 Gy TBI resulted in a reconfiguration of the host immune system by mild lymphodepletion, preferentially of CD8^+^ T cells and B cells. Myeloid cells remained to a great extent, serving as partners for cellular immune responses, especially for antigen presentation. Immature CD4^+^CD8^+^ T cells diminished almost completely in the thymus after 2 Gy TBI but were reconstituted after 11 days, leading to a novel repertoire of unexperienced, antigen-naïve T cells. The important role of endogenous CD4^+^ and CD8^+^ T cells for adoptive T cell therapies has also been pointed out by others 36. Notably, Bogdandi *et al.* showed that suppressive CD4^+^CD25^+^ regulatory T cells and myeloid-derived suppressor cells were significantly diminished by a dose of 2 Gy 37. In addition, Genard *et al.* summarized different studies of TBI on macrophage polarization 38. They identified TBI doses of up to 2 Gy to induce an antitumoral M1 phenotype. Moreover, dendritic cells are activated for antigen presentation by TBI 39. These findings support our conclusion that low-dose TBI induces a competent antitumoral immunologic environment rather than nonselective depletion of essential immune cells.

Moreover, we observed modulation towards a tumor-specific Th1-driven immune response. In line with our findings, Th1 cytokines were shown to be upregulated in splenocytes by single dose irradiation, whereas fractionated TBI fostered Th2 cytokines 14, 37. Gridely *et al.* further showed that irradiation doses in the range of 0.1 to 0.01 Gy already enhanced CD4^+^ T cell responsiveness in C57BL/6 mice 40, demonstrating that optimal dosing of irradiation is critical for adequate immune responses. In contrast, treatment efficacy of CD8^+^ T cell-based ACT increased with irradiation dose 18. This is plausible for CTLs because of their direct lytic ability but cannot be translated to CD4^+^ T cell transfer, as T helper cells strongly depend on additional stimuli, MHC class II-mediated antigen presentation, and phagocytic cell function 41. As a result of the moderate host modulation by our 2 Gy TBI, we found increased numbers of highly active, effector memory Tag-Th1 cells and endogenous CD4^+^ T cells in the pancreatic tumor tissue, enabling a highly effective therapeutic response. A high density of Th1 cells in the tumor is clearly associated with tumor vessel normalization 42, inhibition of metastatic formation 43 and disease control 44.

We further identified enhanced migration of host T cells as well as adoptively transferred Tag-Th1 cells to the liver and lung after 2 Gy TBI. Pneumonitis is a well-known side effect in cancer patients, that is induced by irradiation therapy 45. In contrast, the liver and lung are crucial for antigen presentation and T cell differentiation. Several studies have suggested that the importance of nonlymphatic organs for immune responses might be underestimated. Odoardi *et al.* reported that CD4^+^ T cells were licensed within the lung tissue to enter the central nervous system 46. Additionally, Hamann *et al.* demonstrated that recirculation of T cells through lymphoid tissues diminished following activation, whereas migration into the lung and liver was dramatically upregulated compared to naïve T cells 47. The authors concluded that activated T cells were transiently trapped in the organs where they first arrive, but they remain only in case of an inflamed environment. Our CD3-ImmunoPET studies revealed that this applies to adoptively transferred cells and host T lymphocytes as a consequence of TBI. Notably, local abdominal irradiation can also induce lung inflammation, indicating a systemic rather than direct effect on lung tissue 48. Thus, TBI might have an additional impact on treatment efficacy, especially for lung and liver tumors, as local inflammation processes facilitate persistence of T cells in nonlymphoid tissues.

In search of possible mechanisms that hamper therapeutic efficacy, we identified the spleen as a homing site of a considerable amount of exhausted adoptively transferred and endogenous T cells. T cells circulate from blood into lymph nodes and lymphatic tissue, then back along lymph vessels into the blood to recirculate until they meet their specific antigen or, much more likely, to undergo apoptosis in the spleen 49. Splenectomy led to increased survival of mice treated with combined immunotherapy. Thus, we confirmed the spleen as a negative regulator of antitumoral immune responses. Other researchers also observed reduced tumor-associated macrophage and neutrophil accumulation, and thus delayed tumor growth, after splenectomy in an experimental lung adenocarcinoma mouse model 50. Although splenectomy cannot be regarded as a first-choice treatment option in clinical practice, it might have further implications for irradiation therapy of the lymphoid system. In line with this, two studies revealed beneficial effects of abdominal or spleen irradiation on tumor growth in rats and cancer patients 51, 52.

We further identified enhanced PD-1, LAG-3, and TIM-3 checkpoint molecule expression on splenic T cells after TBI. It has been shown by other groups that TNF and IL-12 foster PD-1 and/or PD-L1 expression 53-55. Thus, the upregulation of immune checkpoints might be related to the TBI-induced inflammatory cytokine release. In our experiments, the additional application of ICB to Th1 cell therapy significantly prolonged the survival of mice with largely progressed tumors, but therapeutic efficacy was still crucially dependent on 2 Gy TBI. This highlights the significance of rational treatment combinations to overcome current immunotherapeutic limitations.

## Conclusion

TBI is a clinically approved and well-established technique. Low-dose TBI enables effective CD4^+^ T cell therapy by favoring Th1-driven antitumoral immunity while synergistically acting host cells are restored, and side effects, such as infectious complications or pulmonary toxicity, can be minimized. It is therefore a promising tool for ACT with CD4^+^ T cells to be integrated into clinical application.

## Supplementary Material

Supplementary figures and table.

## Abbreviations

ACT: adoptive cell therapy
APC: antigen presenting cell
BC: B cells
CAR: chimeric antigen receptor
CT: computed tomography
CTLA-4: cytotoxic T-lymphocyte-associated Protein 4
COMBO: immunotherapy combination
^64^Cu: copper-64
DC: dendritic cells
DOTA: 1,4,7,10-tetraazacyclododecane-1,4,7,10-tetraacetic acid
gran: granulocytes
Gy: gray
ICB: immune checkpoint blockade
LAG-3: lymphocyte activation gene-3
LN: lymph node
mAb: monoclonal antibody
mono: monocytes
NK: natural killer cells
%ID: percent injected (radioactivity) dose
PET: positron emission tomography
PLN: pancreas draining lymph node
PD-1: programmed death protein 1
PD-L1: programmed death ligand 1
RIP1-Tag2: large T cell antigen 2 under the control of the Rat insulin promoter 1
Tag-Th1: large T antigen specific T helper 1 cells
TBI: total body irradiation
TIM-3: T cell immunoglobulin and mucin domain 3
WBC: white blood cells
