# Supplementary material for: Low-dose total body irradiation facilitates antitumoral Th1 immune responses
Source: Theranostics. 2021 Jun 16;11(16):7700–14. doi: 10.7150/thno.61459 (PMC8315067; doi:10.7150/thno.61459)
Supplement: Supplementary file 1 — Supplementary figures and table. [file thnov11p7700s1.pdf]

SUPPLEMENTAL DATA

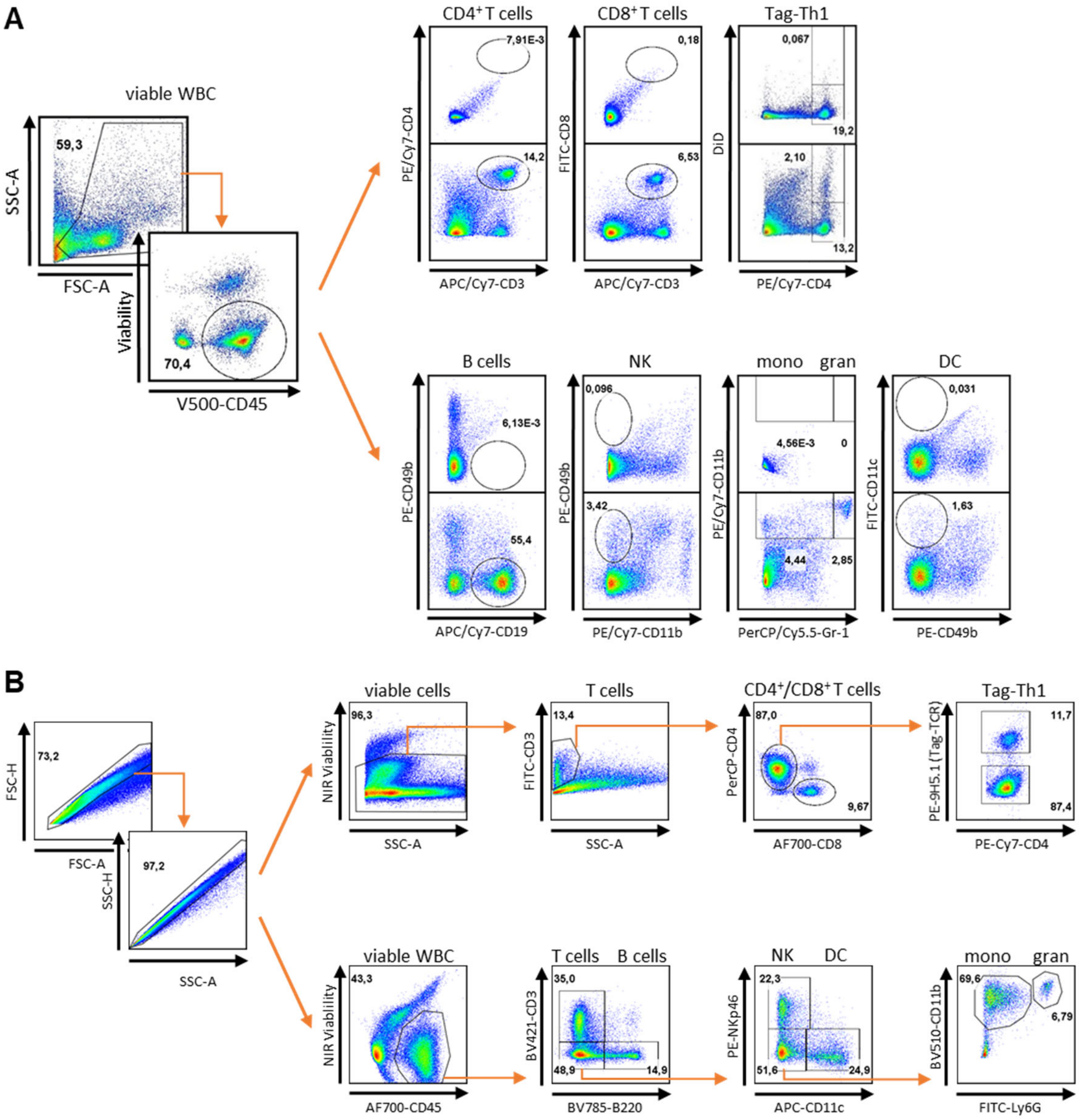

Figure S1 Flow cytometry gating strategy. Exemplary Dot plots illustrate the gating strategy used for flow cytometric analyses of the immune cell composition in C3H mice (A) and tumor-bearing RIP1-Tag2 mice (B). WBC = white blood cells, NK = natural killer cells, mono = monocytes/macrophages, gran = granulocytes, DC = dendritic cells.

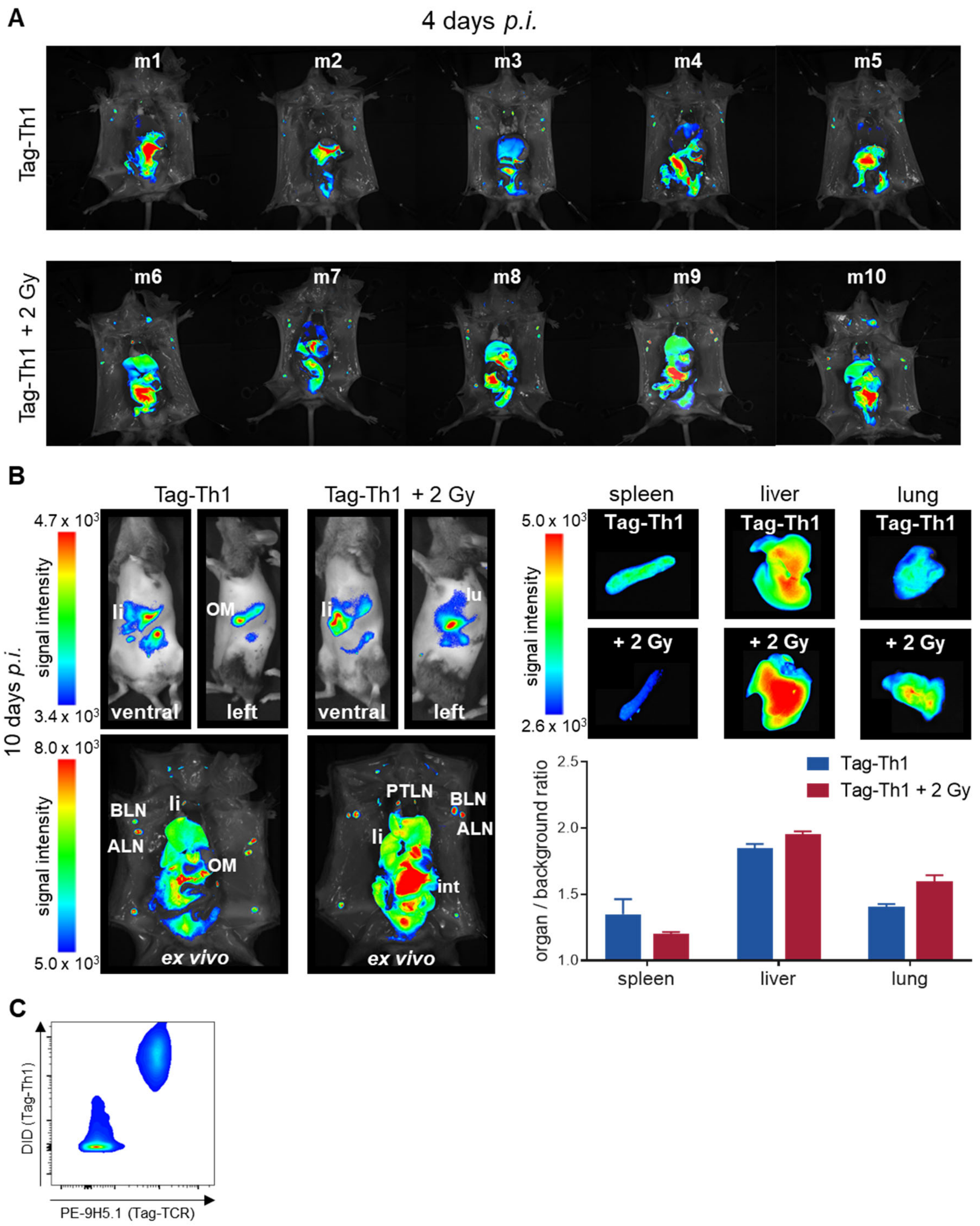

Figure S2 Optical imaging biodistribution of adoptively transferred Tag-Th1 cells in C3H mice. (A) Complete *post-mortem* optical imaging set of all C3H mice 4 days after *i.p* application of 10^7 DID fluorescently-labeled Tag-Th1 cells. 2 Gy TBI was performed 1 day prior to cell administration. (B) *In vivo* (top left), *post-mortem* (lower left), and organ biodistribution quantification (right) of one representative C3H mouse per experimental group 10 days after DID-Tag-Th1 cell application (n = 2 per group). (C) DID-labeled Tag-Th1 cells isolated from lymph nodes were double-stained with a Tag-TCR targeting fluorescently-labeled mAb (9H5.1) to confirm specific recognition by both labeling methods via flow cytometry.

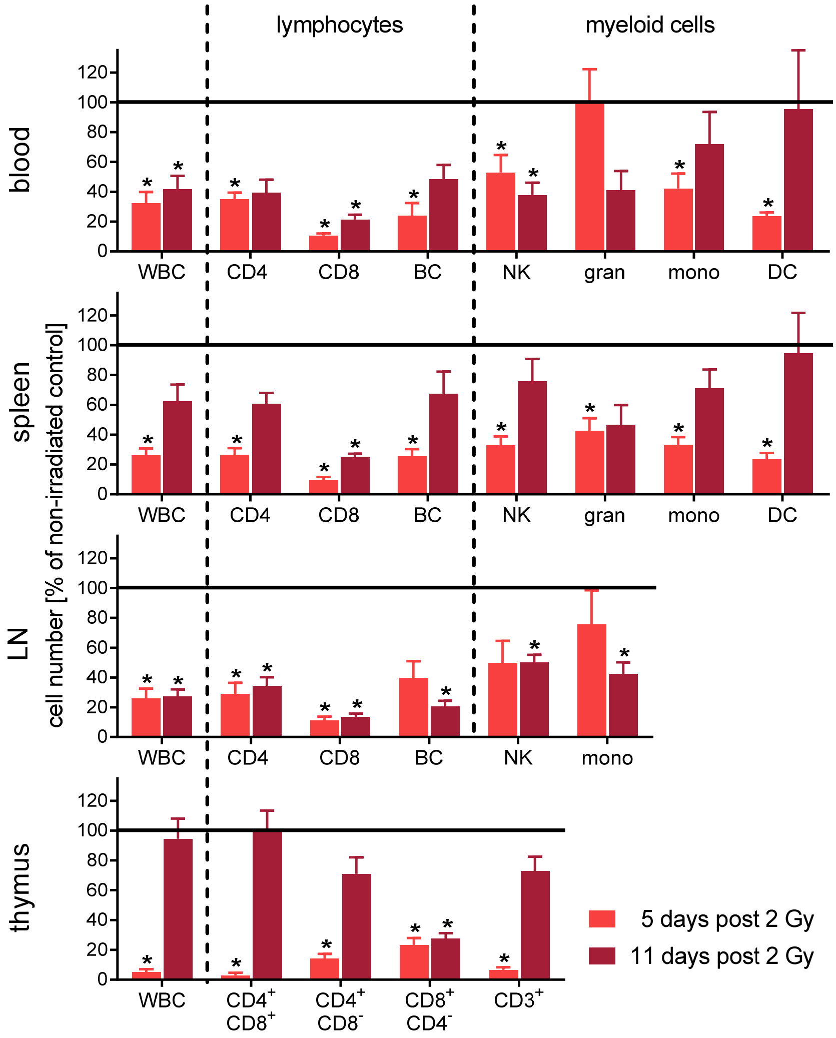

Figure S3 Low-dose TBI induces differential cell number alterations of lymphoid and myeloid cell populations in blood and lymphatic organs. Multicolor flow cytometric analyses 5 and 11 days post-2 Gy TBI of the main immune cell populations in the blood, spleen, extraperitoneal lymph nodes (LN) and thymus (n = 5 per group). Lymphocyte populations were more affected by 2 Gy TBI compared to myeloid cells. Absolute cell numbers per organ were calculated by total WBC count of each organ (or per µL blood) x cell subset fraction of viable $CD45^+$ cells and stated as the percentage of untreated controls (mean±SEM). Cell subsets were classified as white blood cells (WBC), $CD3^+CD4^+$ T cells (CD4), $CD3^+CD8^+$ $CD8^+$ T cells (CD8), $CD19^+$ B cells (BC), $CD49b^+$ natural killer cells (NK), $CD11b^+Gr\text{-}1^{High}$ granulocytes (gran), $CD11b^+Gr\text{-}1^{low}$ monocytes (mono), and $CD11c^+$ dendritic cells (DC).

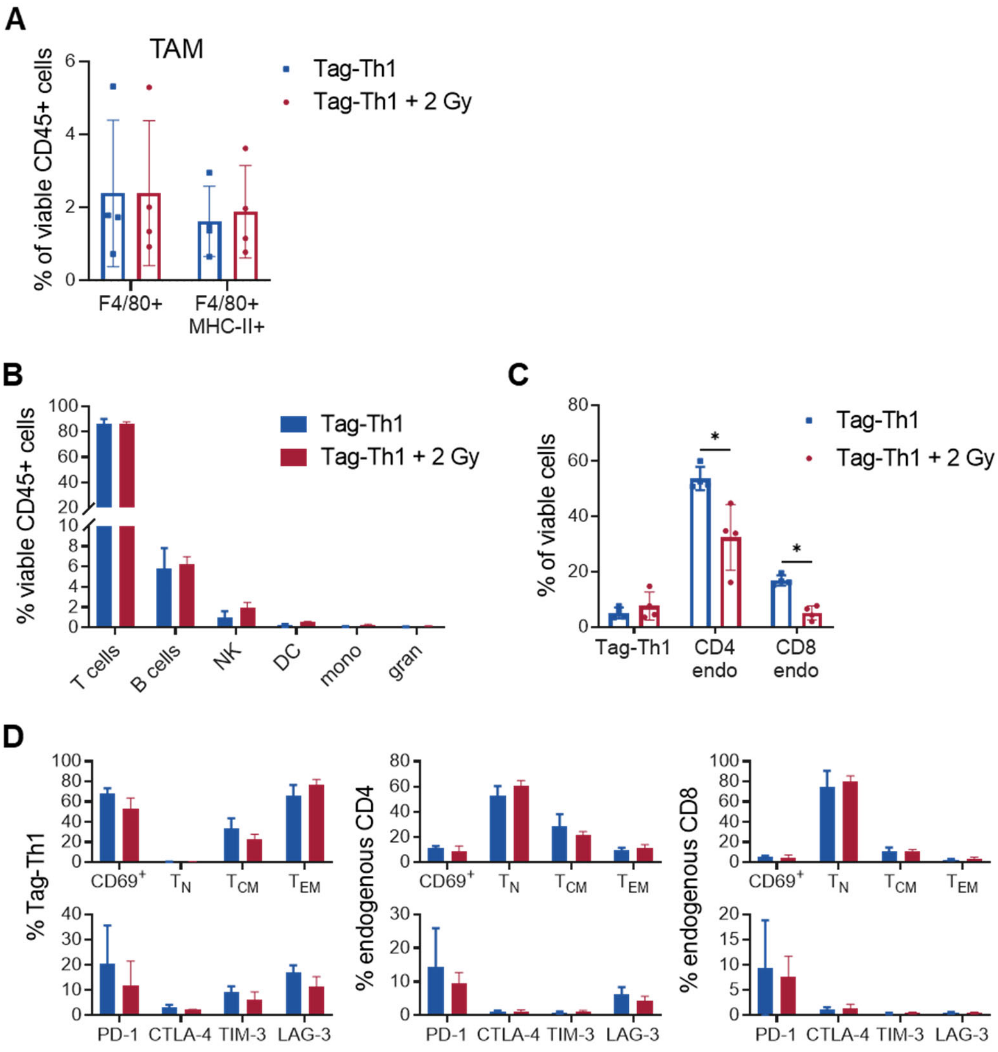

Figure S4 Immune cell and Tag-Th1 cell biodistribution in the pancreatic tumor tissue and pancreas draining lymph nodes of RIP1-Tag2 mice (n = 4 per group)**.** (A) Tumor associated macrophages (TAM, CD11b+F4/80+) of the pancreas and (B) immune cell composition of the pancreas draining lymph node of nonirradiated and 2 Gy TBI mice analyzed 10 days after Tag-Th1 cell administration (11 days post-2 Gy TBI) by multicolor flow cytometry. Cell subsets were classified as CD3$^+$ T cells, CD19$^+$ B cells, NKp46$^+$ NK cells (NK), CD11c$^+$ dendritic cells (DC), CD11b$^+$Ly6G$^-$ macrophages/monocytes (mono), and CD11b$^+$Ly6G$^+$ granulocytes. (C) Lower numbers of host CD3$^+$CD4$^+$ T cells (CD4 endo) and CD3$^+$CD8$^+$ T cells (CD8 endo) were detected in Tag-Th1 and 2 Gy TBI treated mice, while Tag-Th1 cells remained stable. (D) Adoptively transferred and host T cells of the pancreas draining lymph node were analyzed for activation status (CD69$^+$), phenotypic differentiation (CD44$^-$CD62L$^+$ naïve (T$_{N}$), CD44$^+$CD62L$^+$ central memory (T$_{CM}$), CD44L$^+$CD62L$^-$ effector memory (T$_{EM}$) T cells) and expression of immune checkpoint molecules.

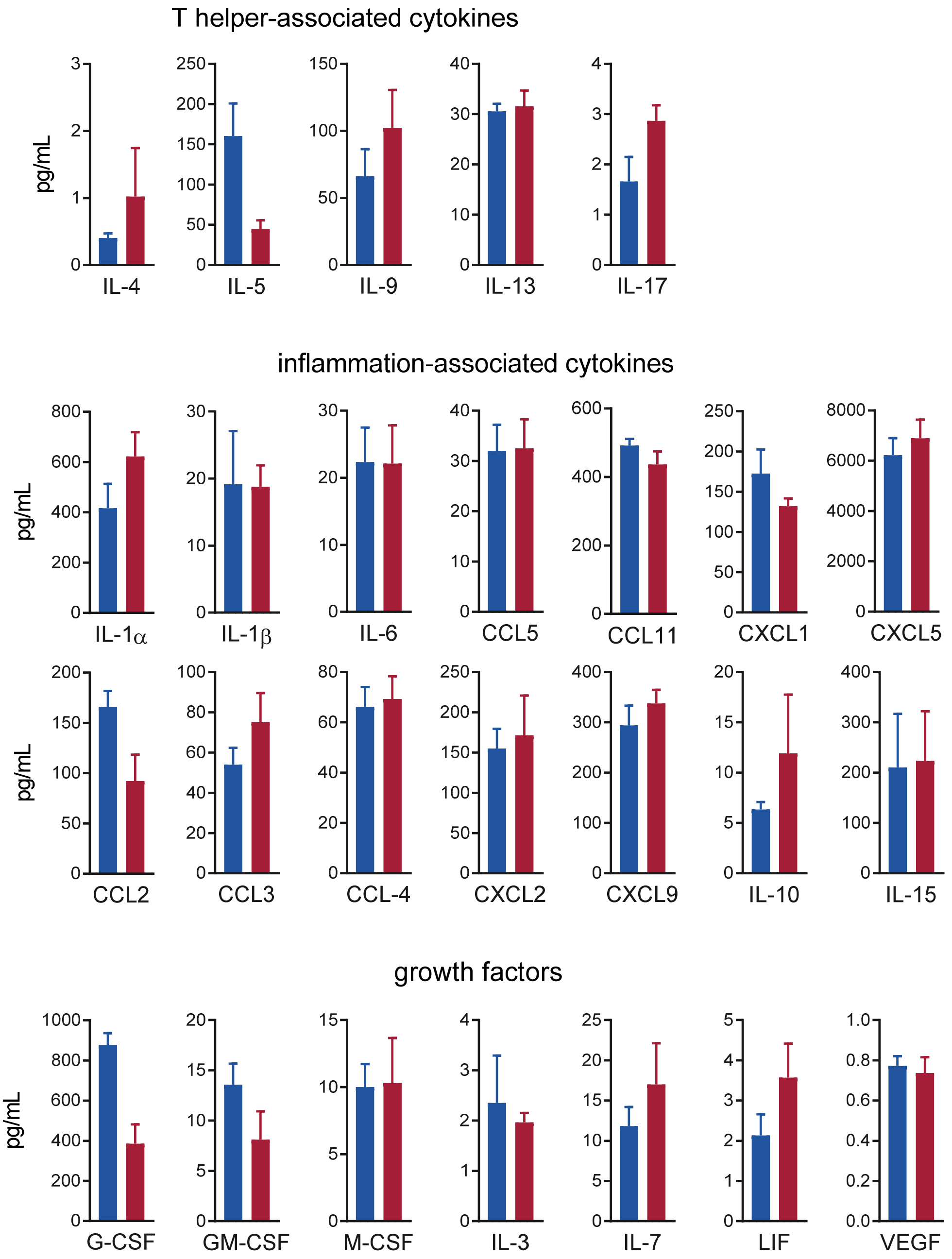

Figure S5 Blood cytokine levels in tumor-bearing RIP1-Tag2 mice. T helper cell (Th1 is shown in Fig. 4 E) or inflammation-associated cytokine levels and growth factor expression levels of tumor-bearing RIP1-Tag2 mice 4 days after Tag-Th1 cell administration and 5 days after 2 Gy TBI (or sham irradiation) (n = 4-5 per group).

Table S1 Ratio of Tag-Th1 to various host immune cell populations in blood, spleen, and lymph nodes 4 days (above) and 10 days (below) after Tag-Th1 cell application. 2 Gy TBI induced increase by <1.5-fold increase (grey), 1.5 - 2.9-fold (light green), 3.0 - 5.0-fold (green), >5-fold (dark green). Significant differences between the non-irradiated (control) and 2 Gy TBI group were marked * and in bold. BC = B cells, NK = natural killer cells, gran = granulocytes, mono = monocytes, DC = dendritic cells.

	Ratio Tag-Th1 to	blood		spleen		lymph nodes	
		control	2 Gy	control	2 Gy	control	2 Gy
4 days	WBC	0.35 (± 0.08)	**1.35 (± 0.52)***	0.30 (± 0.10)	**1.12 (± 0.16)***	0.25 (± 0.07)	**1.17 (± 0.46)***
	CD4	2.24 (± 0.48)	**6.83 (± 1.34)***	3.71 (± 1.52)	**13.2 (± 2.4)***	0.43 (± 0.12)	**1.76 (± 0.74)***
	CD8	3.90 (± 0.92)	**40.1 (± 13.2)***	6.26 (± 2.88)	**62.7 (± 13.0)***	0.89 (± 0.24)	**9.60 (± 4.52)***
	BC	1.10 (± 0.33)	7.78 (± 6.70)	0.52 (± 0.16)	**2.02 (± 0.32)***	3.44 (± 1.16)	**10.5 (± 3.7)***
	NK	13.0 (± 3.7)	**30.1 (± 10.0)***	9.77 (± 4.28)	**28.8 (± 4.7)***	21.0 (± 6.6)	**49.9 (± 11.4)***
	gran	8.55 (± 3.94)	8.55 (± 2.51)	15.7 (± 8.7)	**46.8 (± 21.6)***		
	mono	2.67 (± 1.02)	**7.77 (± 2.72)***	4.01 (± 1.48)	**13.9 (± 4.5)***	92.4 (± 35.2)	156 (± 57)
	DC	71.7 (± 13.0)	**338 (± 99)***	15.7 (± 6.9)	**65.0 (± 15.3)***		

	Ratio Tag-Th1 to	blood		spleen		lymph nodes	
		control	2 Gy	control	2 Gy	control	2 Gy
10 days	WBC	0.20 (± 0.05)	**0.76 (± 0.12)***	1.87 (± 0.31)	**3.57 (± 1.05)***	0.35 (± 0.11)	**0.97 (± 0.22)***
	CD4	1.17 (± 0.21)	**4.44 (± 0.67)***	13.3 (± 3.4)	**24.1 (± 3.1)***	0.63 (± 0.24)	**1.32 (± 0.32)***
	CD8	2.24 (± 0.57)	**14.9 (± 4.1)***	28.9 (± 7.4)	**125 (± 13)***	1.17 (± 0.40)	**6.43 (± 1.27)***
	BC	0.83 (± 0.37)	**2.48 (± 0.57)***	3.38 (± 0.65)	6.24 (± 2.37)	4.51 (± 0.65)	**16.4 (± 3.8)***
	NK	4.69 (± 1.43)	**19.3 (± 2.9)***	56.8 (± 7.4)	91.1 (± 27.4)	21.4 (± 5.1)	32.7 (± 11.4)
	gran	1.50 (± 0.57)	8.23 (± 5.62)	65.9 (± 14.5)	199 (± 126)		
	mono	1.92 (± 0.61)	**4.32 (± 0.84)***	39.1 (± 5.9)	65.4 (± 21.5)	75.1 (± 12.9)	**129 (± 36)***
	DC	28.9 (± 7.8)	**48.3 (± 24.5)***	113 (± 9)	165 (± 78)		
